# Community structure of pollinating insects and its driving factors in different habitats of Shivapuri‐Nagarjun National Park, Nepal

**DOI:** 10.1002/ece3.8653

**Published:** 2022-03-01

**Authors:** Urmila Dyola, Chitra Bahadur Baniya, Pushpa Raj Acharya, Pradip Subedi, Anjeela Pandey, Kumar Sapkota

**Affiliations:** ^1^ Central Department of Zoology Institute of Science and Technology Tribhuvan University Kirtipur Nepal; ^2^ Department of Zoology Patan Multiple Campus Lalitpur Nepal; ^3^ Central Department of Botany Tribhuvan University Kirtipur Nepal; ^4^ Central Campus of Science and Technology Faculty of Science and Technology Mid‐West University Surkhet Nepal; ^5^ 7788 School of Natural Sciences Macquarie University North Ryde NSW Australia

**Keywords:** community composition, open trail, pollinators, Shivapuri‐Nagarjun National Park, species richness

## Abstract

Insect pollinators are important means for a stable ecosystem. The habitat types play a crucial role in the community composition, abundance, diversity, and species richness of the pollinators. The present study in Shivapuri‐Nagarjun National Park explored the species richness and abundances of insect pollinators in four different habitats and different environmental variables in determining the community composition of the pollinators. Data were collected from 1,500 m to 2,700 m using color pan traps and hand sweeping methods. Non‐Metric Multidimensional Scaling (NMDS) and Redundancy Analysis (RDA) were conducted to show the association between insect pollinators and environmental variables. The results firmly demonstrated that species richness and abundances were higher (158) in Open trail compared to other habitats. The distribution of the pollinator species was more uniform in the Open trail followed by the Grassland. Similarly, a strong positive correlation between flower resources and pollinators' abundance (R^2^ = .63, P < .001) was found. In conclusion, the Open trail harbors rich insect pollinators in lower elevation. The community structure of the pollinators was strongly influenced by the presence of flowers in the trails.

## Graphical abstract

We sampled the pollinators along the elevation gradients of Shivapuri–Nagarjun National Park in four types of habitats; forest trail, grassland, trails of managed habitat, and open trail of the forest. Open trail of the forest that hold more floral resources was high in the species richness and abundance of insect pollinators that decrease with the increase of elevation. A strong positive correlation between flower resources and pollinator's abundance was found.

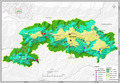

## 1 INTRODUCTION

Pollinators are important agents for a stable ecosystem (De Groot et al., 2010; Kremen, 2008). They enhance pollination for both wild and cultivated flowering plants and help humans to increase agricultural production (Corbet et al., 1991; Kevan et al., 1990; Widhiono et al., 2016). These insects also benefit the economic, aesthetic, and cultural aspects of mankind (Gill et al., 2016). In general, social and solitary bees, butterflies and moths, beetle and flies account as dominant pollinators (Vanbergen & Initiative, 2013; Wojcik, 2021).

Most of the cultivated plants around the world are pollinated by bees (56.5%), flies (19%), and butterflies (4%) (Bashir et al., 2019). Honeybees are better‐known bees for pollination in comparison with wild pollen bees (Losey & Vaughan, 2006; Potts et al., 2010) while flies and butterflies are the least known as pollinating insects (Jennersten, 1984; Larson et al., 2001). Several studies show that there is decline in pollinators globally (Carvalheiro et al., 2013; Dirzo et al., 2014). Especially, the population of bumblebees (Cameron et al., 2011; Fitzpatrick et al., 2007) and butterflies (Van Swaay et al., 2010; Warren et al., 2001) is shrinking in the world due to natural as well as anthropogenic threats. The major drivers of pollinator loss are recognized as habitat loss, landscape modification, intensification in agriculture, and even climate change (Kearns et al., 1998; Kovács‐Hostyánszki et al., 2017; Potts et al., 2010). Hence, maintaining pollinator diversity in the given landscape requires an understanding of a clear pattern of pollinator diversity along with the habitat types.

The species richness, diversity, distribution, and community structure of pollinators depend upon the local environment (Neumüller et al., 2020; Williams et al., 2010). Availability of flower resources, humidity, and temperature need to be taken into account especially while comparing the pollinators among habitats (Neumüller et al., 2020). The activity of pollinators is strongly correlated with air temperature, plant species richness (Hudewenz et al., 2012), and humidity (Pellissier et al., 2010). Bees and butterflies prefer warmer temperatures than flies (Kühsel & Blüthgen, 2015). Furthermore, elevation also determines the abundance and community structure of insect pollinators (Adedoja et al., 2020). There is an interesting distribution pattern among different groups of insect pollinators. Hymenoptera is the dominant pollinator in the lowland while Lepidoptera and Diptera dominate the high land (Warren et al., 1988). This kind of distribution in a range of habitats is probably for fulfilling their ecological requirements and these ecological necessities are mostly species or guild specific (Proesmans et al., 2019). For instance, bees prefer to forage the flowering plants close to their nesting area (Gathmann & Tscharntke, 2002; Greenleaf et al., 2007). They also construct the nest in deadwood (Sydenham et al., 2016) and the sun‐exposed soil ground (Everaars et al., 2011). However, hoverflies and butterflies fly away from the egg‐laying areas for foraging and do not construct the nest. Aphidophagous hoverflies such as *Episyrphus*, *Sphaerophoria* depend on agricultural habitat (Jauker et al., 2009; Pinheiro et al., 2015), while saproxylic hoverflies (*Xylota*) are benefitted from the forest (Reemer, 2005). Similarly, butterflies can make a flight over greater distances (Herrera, 1987). They make such range of flight in search of different kinds of flowers for pollen (Gilbert, 1972) and nectar (Tiple et al., 2005). Additionally, oviposition‐plant's location signifies habitat selection for smaller and less mobile butterflies, such as the ‘blues’ while the larger butterflies like *Erebia epipsodea* and *Colias* probably have large ranges of the search for their widespread ovipositing plants (Sharp et al., 1974). The above examples show that pollinators share different habitats and hence the habitat types potentially impact the community composition, abundance, diversity, and species richness of the pollinators.

Many studies have evaluated how environmental factors influence pollinator composition. However, the effects of many environmental factors on pollinator composition can be very different among climate/vegetation zones (Neumüller et al., 2020; Senapathi et al., 2017). Shivapuri‐Nagarjun National Park (SNNP) lies in the southern foot of the Himalayas, a mountainous area with a complex landscape. The unique landscape of the park could harbor interesting pollinator fauna and number of environmental factors may differently influence their composition. So, it is necessary to test the roles of these factors on pollinators in SNNP. The general understanding about natural habitat is, if freed from pesticides, it probably possesses more diversity than the managed habitat. However, the habitat heterogeneity would also affect the pollinator community as the interaction between plant–pollinator is specific (Oliver et al., 2010; Rundlöf et al., 2008; Weibull et al., 2000).

We examined species richness, abundance, and the community composition of insect pollinators in different habitats along the elevation gradient. We hypothesized that there is an effect of habitat types in the diversity, distribution, and composition of pollinating insects. Our research questions were as follows: ‘What is the distribution pattern of pollinating insects? Which environmental factors significantly influence this distribution pattern, diversity, and community composition of pollinators?’.

## 2 MATERIALS AND METHODS

### 2.1 Study area

**FIGURE 1:**
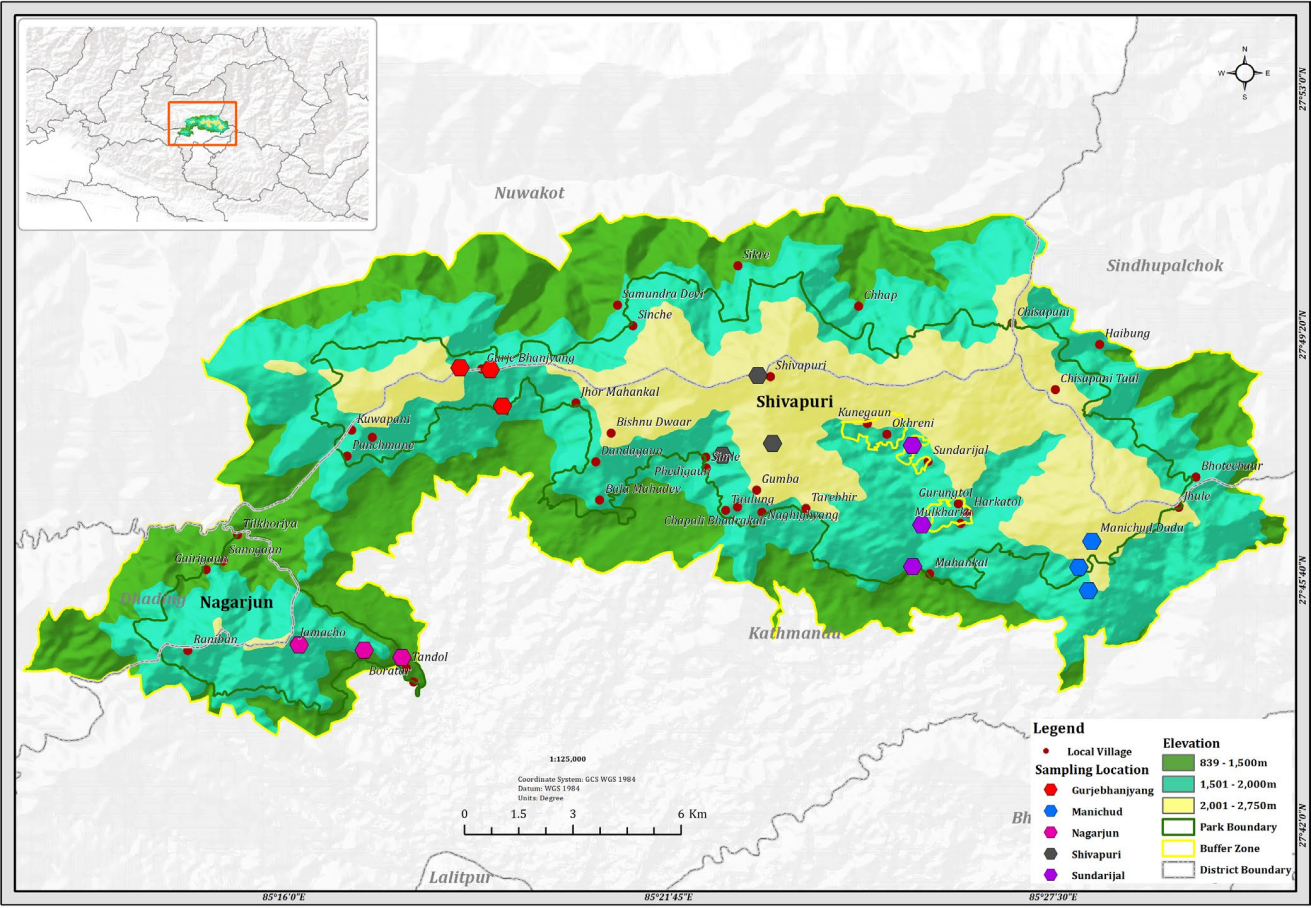
Map of study area showing the location of sampling sites

Shivapuri‐Nagarjun National Park (Figure 1) is the only protected area in the mid‐hill region of Nepal. The Park covers an area of 159 square kilometers with a buffer area of around 118.61 square km. It lies within 27°45′ to 27°52′N Latitude and 85°16′ to 85°45′E Longitude. It has an altitude ranging from 1,360 m to 2,732 m above sea level. The Park is rich in freshwater resources with abundant biodiversity and cultural heritage (SNNP, 2017). The Park possesses a subtropical to warm temperate weather with an average maximum and minimum temperature of 19.9°C and 11.15°C, respectively, and the mean annual precipitation of 236.5 mm (Climatic data from 1985 to 2017 AD, Department of Hydrology and Meteorology/DHM).

The mountainous topography of SNNP with steep slopes of >30% uses a pattern that constitutes 74.45% forest, 20.45% shrubland, 3.22% grassland, 1.80% cultivated area, and 0.055% other features (SNNP, 2017). Different forest types are present under different altitudes of the Park: Lower Mixed Hardwood, Chir pine Forest, Oak Forest, and Upper Mixed Hardwood Forest (Amatya, 1993). The major tree species are *Schima wallichii*, *Alnus nepalensis*, *Castanopsis* spp., *Myrica esculanata*, *Madhuca indica*, *Pinus roxburghii*, *Pyrus pashia*, *Aesculus* sp, *Acer* sp., *Salix* sp., *Lithocarpus* sp, *Ilex dipyrena*, etc.

### 2.2 Sampling sites

We identified five representative study sites across the SNNP, namely, Sundarijal (27°.77′N Latitude and 85°.42′E Longitude), Shivapuri (27°.79′N Latitude and 85°.37′E Longitude), Gurjebhanjyang (27°.81′N Latitude and 85°.31′E Longitude), Nagarjun (27°.74′N Latitude and 85°.27′E Longitude), and Manichud (27°.77 N Latitude and 85°.46′E Longitude). We selected these five sites as they represent the chosen habitat and elevation for survey. We sampled all five sites in three different elevations; a lower transect occurred between 1,500 m and 1,700 m, middle transect at 1,800 m–1,900 m, and upper transect at 2,000 m–2,700 m. These elevations included four different habitats: Forest trail (FT), Grassland (GL), the trail of the Managed habitat (MH), and the Open trail of the forest (OT). In each elevation, five transects, each of 100 m × 5 m with 100 m inter‐transect distance, were fixed. So, a total of 75 transects were surveyed in the study sites. The number of transects in FT, GL, OT, and MH were 33, 14, 15, and 13, respectively (Table 1).

**TABLE 1:** Number of sampling transects in four different habitats along the elevation gradient of Shivapuri‐Nagarjun National Park, Nepal

Habitat	Elevation (meter)	Number of transects	Description
Forest trail	1,500–1,700	11	Forest trail is denoted as the forest with canopy coverage of more than 70% with a walking trail of 15 m width
	1,800–1,900	12	
	2,000–2,700	10	
Grassland	1,500–1,700	3	Grassland habitat is herb‐dominated in the transect with sparsely located trees
	1,800–1,900	6	
	2,000–2,700	5	
Open trail	1,500–1,700	4	Open trail habitat of the forest means an area with no tree coverage within 15 m width on either side of the survey transect
	1,800–1,900	5	
	2,000–2,700	6	
Managed habitat	1,500–1,700	7	The Open trail of managed habitat is the transect along the walking trail of the managed habitat with no canopy tree within 15 m width of the trail that incorporates the buffer zone area of the National Park. The area is a human settlement with cultivated lands. The major crops cultivated during spring are mustard and coriander while mustard, buckwheat, balsam apple, and squash are in autumn
	1,800–1,900	2	
	2,000–2,700	4	

Note

The fourth column of the table describes the characteristics of each habitat.

### 2.3 Pollinators' sampling

Pollinators here mean flower‐visiting and nectar‐feeding insects. The transect line was fixed in walking trail of Forest, Open forest, and Managed habitat, while in the Grassland transects were drawn at the edge and the middle of the Grassland with 20‐m inter‐transect distance (Stanley, 2013). The survey was performed in the spring and autumn seasons for the consecutive years 2018 and 2019. In each season, the pollinators were sampled for 3 consecutive days in sunny weather between 9 am and 4 pm (Pollard & Yates, 1994). The pollinators were collected using hand sweeping and color pan traps. White, yellow, and blue color pans were used for insect sampling. During each sampling day, a transect walk of 30 min was made along the 100 m of the trail in the Open trail of the forest, Forest trail, and trail of the Managed habitat, while in Grassland the transect walk was made at the edge and the middle (Stanley et al., 2013). The transect walk method was used to sample butterflies (Pollard & Yates, 1994), bumblebees (Fussell & Corbet, 1992), hoverflies, and other bees (Proesmans et al., 2019). During the walk, all insect pollinators, which fed on flowers within 2 m of the observer, were captured, except for those that could be identified to a species level in the field (Neumüller et al., 2020). Unidentified insect pollinators were collected in separate vials, transferred in the icebox, and brought to the laboratory for identification.

Similarly, colored pan traps were deployed in each transect. This method aids in the simultaneous sampling of multiple locations, coverage of a large number of sites, and is the efficient method of bee sampling (Nielsen et al., 2011; Westphal et al., 2008). The pans were plastic bowls of about 15 cm in diameter and painted with non‐toxic three different colors; white, yellow, and blue (LeBuhn et al., 2003). Each pan was attached to a post using a metal clamp adjusting the bowl in the rim. The pan was filled with 400 ml of detergent water. Three posts were deployed at a 100‐m distance, 20 m apart from each other. The traps were visited for collecting the fallen insects after 24 h and were transferred in labelled vials with 70% ethanol.

### 2.4 Survey of flower resources

The survey of flower resources was carried out in the spring and autumn season during the pollinator survey in the same transect. We made five quadrats of 10 m × 5 m in each sampling transect. For an estimation of abundance flower resources, we scanned insect pollinating herbs and shrubs in each quadrat and identified the genus and species. The cover of flower resources in each quadrat was ranked between 1 and 6 (Szigeti et al., 2016). Rank: very scarcely = 1, scarce = 2, more or less scarce = 3, more or less abundance = 4, abundance = 5, and extremely abundance = 6. The abundance of flower resources for each sampling transect was calculated as $\text{FLOWER}=\sum_{I=1}^{N}t.taxon_{i}\left(t=\text{mean of the rank of flowering plant of each transect}\right)$.

### 2.5 Humidity and temperature

The humidity and temperature during each sampling time were measured with a digital Thermo‐hygrometer (HTC‐2).

### 2.6 Identification of insect pollinators

Unidentified insect pollinators in the field were identified to species level in the laboratory using relevant keys. Bingham (1897), Tadauchi and Matsumura (2007), Williams et al. (2010), Bodlah et al. (2016), Aslam et al. (2017), Ngat et al. (2017), and Kumari et al. (2018) for bee specimens. Brunetti (1923), Thompson and Ghorpade (1992), Ghorpadé (1994), Ghorpadé (2019), Claussen and Weipert (2003), Sengupta et al. (2017), Hassan et al. (2019), Hassan et al. (2020), Sankararaman et al. (2020) for hoverflies, and Smith (2011) for butterflies specimen's identification.

### 2.7 Statistical analysis

For each habitat type, we decided the cumulative species richness and species abundance across all samplings and assembled the community matrix. The species accumulation curves were plotted by using the package ‘vegan’, function specaccum to test the adequacy of sampling effort. Pollinator's richness and abundance were compared between different habitats, using linear mixed‐effect models with habitats as predictor variables and pollinators as response variables. The statistical analysis was performed in Program R (R Core Team, 2021). Kruskal–Wallis test was carried out to find significance because data were not normal and different in the number of habitat types.

PAST: Paleontological Statistics (Hammer et al., 2001) Version 3.17 computed the diversity indices. Random matrices with two samples were generated, each with the same row and column totals as in the original data matrix, which provided the significance of diversity between groups.

Pollinator's community compositions of different habitats (FT, GL, OT, and MH) were analyzed by Non‐metric Multidimensional Scaling (NMDS) of the abundance data employing the function ‘metaMDS’ which is incorporated in the statistical package ‘vegan’ (Oksanen et al., 2013) and NMDS result with sample plots of different abundance scores were fitted with different habitats using the package ‘ggplot2’ (Wickham et al., 2016).

NMDS was followed by statistical analyses: Adonis (Permutational Multivariate Analysis of variance), ANOSIM (Analysis of Similarities), and SIMPER (Similarity Percentage Analysis).

Adonis was carried out following NMDS to analyze statistically if the pollinator's community differs between the habitats. It provides the *p*‐value to determine the statistical significance. ANOSIM, on the other hand, was used to determine if the differences of pollinator's community between the habitats are significant. In addition to the significant difference tests, SIMPER analysis was used to identify those species that contributed the most to the observed pollinator's community differences (Clarke & Gorley, 2001).

To find relations between the environmental variables and the species composition, ordinations were performed on insect pollinators. For the pollinator's community of 15 most abundance species, five from each group; bee, butterfly, and hoverfly (Supplementary 1), a Detrended Correspondence Analysis (DCA) was carried out to decide whether unimodal or linear ordination methods were appropriate (Lepš & Šmilauer, 2003). DCA analysis projected a gradient length of 2.5 indicating Redundancy Analysis (RDA) ordination as an appropriate process. Environmental variables were backward selected (*p* < .05) and a Monte Carlo permutation test with 999 iterations was used to assess the significance of the ordination.

NMDS, RDA, and all of the three procedures (Adonis, ANOSIM, and SIMPER) were carried out in Program R (R Core Team, 2021) using the package ‘vegan’ (Oksanen et al., 2013). Venn diagrams showing the species distribution between the habitats were performed in Program R (R Core Team, 2021) by using the package ‘VennDiagram’ employing the function draw.quad.venn.

## 3 RESULTS

### 3.1 Insect pollinators in Shivapuri‐Nagarjun National Park

**FIGURE 2:**
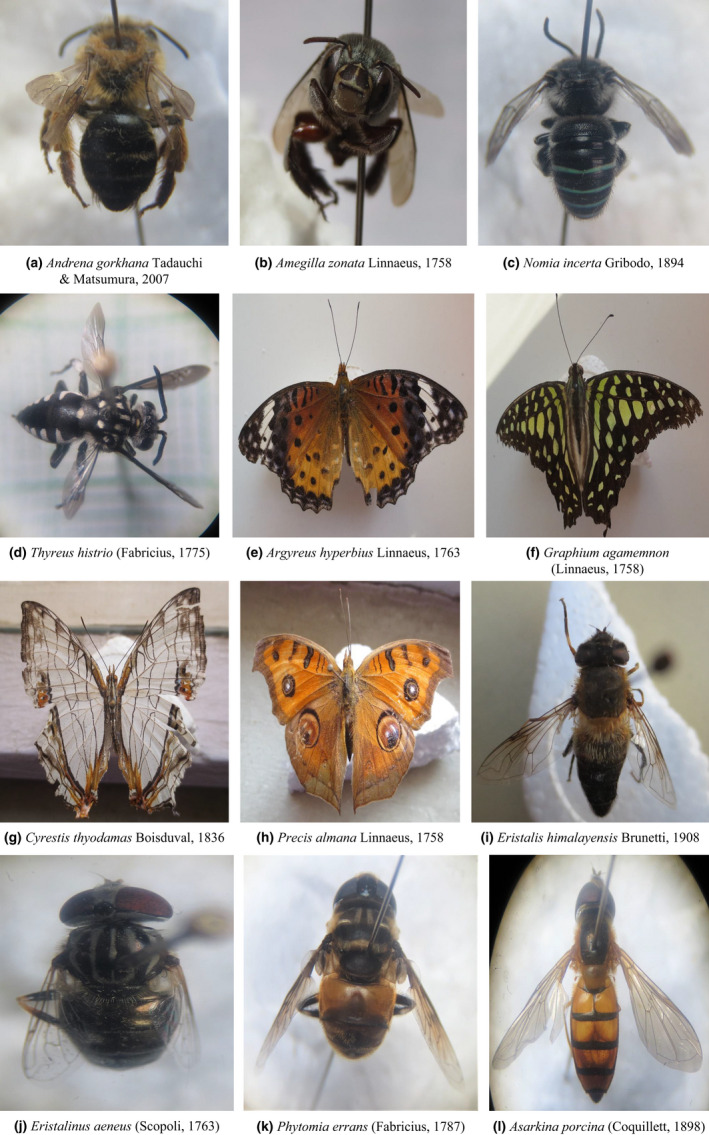
A range of insect pollinators collected from four different habitats of Shivapuri‐Nagarjun National Park (Bees: a–d, Butterflies: e–h, and Hoverflies: i–l). (a) *Andrena gorkhana* Tadauchi and Matsumura, 2007; (b) *Amegilla zonata* (Linnaeus, 1758); (c) *Nomia incerta* Gribodo, 1894; (d) *Thyreus histrio* (Fabricius, 1775); (e) *Argyreus hyperbius* Linnaeus, 1763; (f) *Graphium agamemnon* (Linnaeus, 1758); (g) *Cyrestis thyodamas* Boisduval, 1836; (h) *Precis almana* Linnaeus, 1758; (i) *Eristalis himalayensis* Brunetti, 1908 (j) *Eristalinus aeneus* (Scopoli, 1763); (k) *Phytomia errans* (Fabricius, 1787); (l) *Asarkina porcina* (Coquillett, 1898)

**FIGURE 3:**
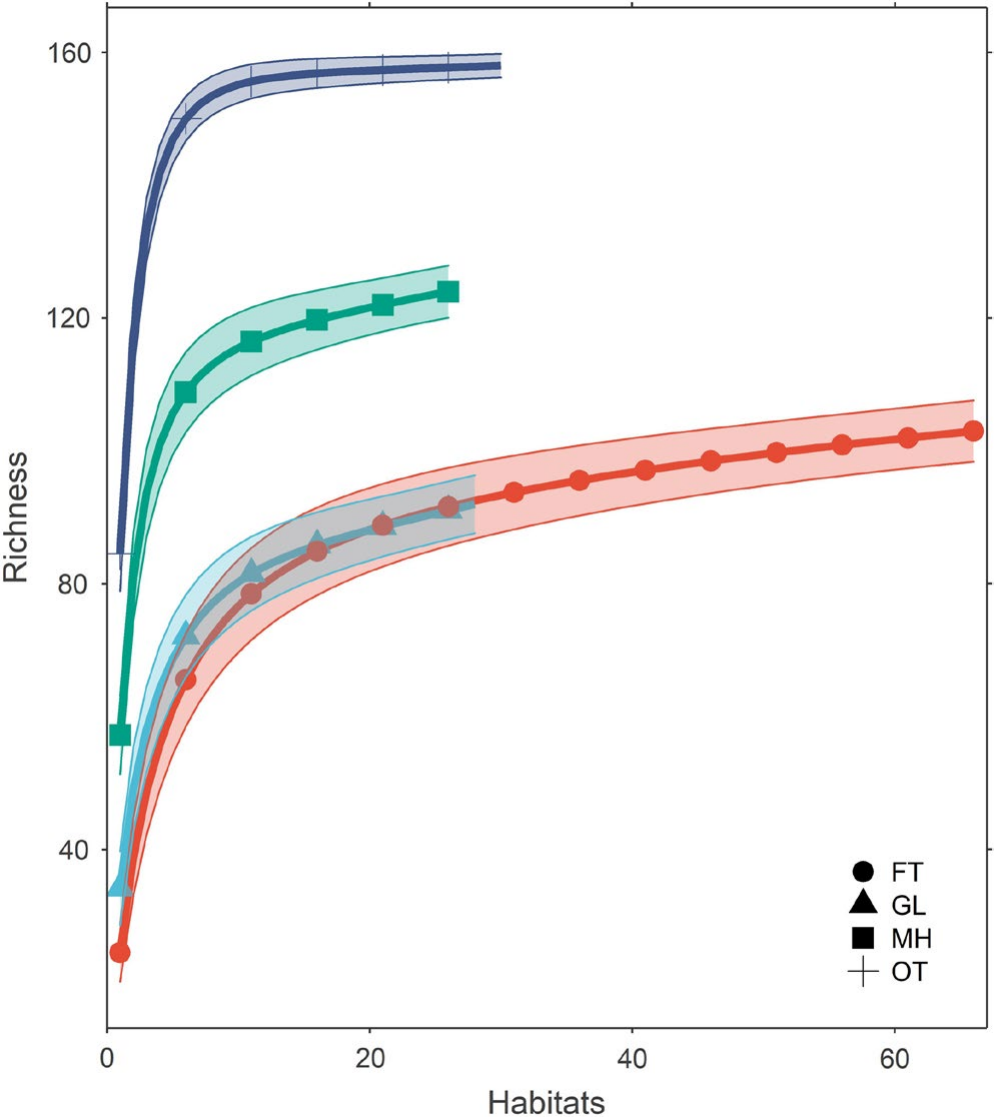
Species accumulation curve for pollinator's community in four different habitats of Shivapuri‐Nagarjun National Park. Each line represents a different habitat. Habitat: FT‐ Forest trail with canopy, GL‐ Grassland, MH‐ Managed habitat, and OT‐ Open trail of the forest without canopy

During the total sampling period, 8720 insect pollinators were caught, belonging to 167 different species (see Supplementary 1) from both pan traps (1,339) and sweeping net (7,381); the butterfly with the most dominant species (48.50%) followed by a bee (29.94%) and hoverfly (21.56%). Representative 12 insect pollinators collected from different habitats of SNNP are shown in Figure 2. Sixty flower herbs and shrubs were recorded from both sampling seasons in both years (Supplementary 2). The abundance of flower resources varied between habitats, elevations, and seasons (see Supplementary 3). Species accumulation curves for pollinators showed saturation in all habitat types indicating adequate sampling effort (Figure 3).

### 3.2 Abundance and species richness of insect pollinators in different habitats

**FIGURE 4:**
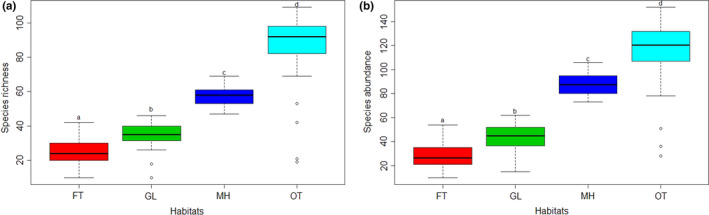
Species richness (a) and species abundance (b) of insect pollinators in four different habitats of Shivapuri‐Nagarjun National Park. Horizontal line across each box represents median, whiskers show the data range, and circles indicate outliers. Habitat: FT‐ Forest trail with canopy, GL‐ Grassland, MH‐ Managed habitat, and OT‐ Open trail of the forest without canopy

Species richness and abundances were higher in Open trail than in other habitats (Figure 4). There is a significant difference in species richness (Kruskal–Wallis test, *χ* ^2^ = 104.96, df = 3, *p* < .001) and abundance among habitats (Kruskal–Wallis test, *χ* ^2^ = 110.16, df = 3, *p* < .001).

### 3.3 Diversity and Distribution pattern of insect pollinators

Diversity indices in alpha level (Species richness, Shannon index) show that the Open trail was comparatively more diverse, followed by the Managed habitat. The distribution of the pollinator species was much even in the Open trail followed by Grassland. The species richness and Shannon index were significant (*t*‐test, *p* < .05) in all of the habitat types. Similarly, similar evenness was observed in Forest trail and Grassland (Table2).

**TABLE 2:** S: Species richness, H: Shannon–Weiner, J: Pielou's evenness, D: Dominance

		S	H	J	D
Habitat	MH	124^a^	4.30^a^	0.59^a^	0.024^a^
	FT	103^b^	4.19^b^	0.64^b^	0.020^b^
	GL	92^c^	4.09^c^	0.65^b^	0.022^a^
	OT	158^d^	4.85^d^	0.81^c^	0.010^c^

Note

On a given column, values followed by the different superscripted letter (a, b, c, d) are significantly different at *p* < .05 by the *t*‐test comparing diversity in PAST software.

**FIGURE 5:**
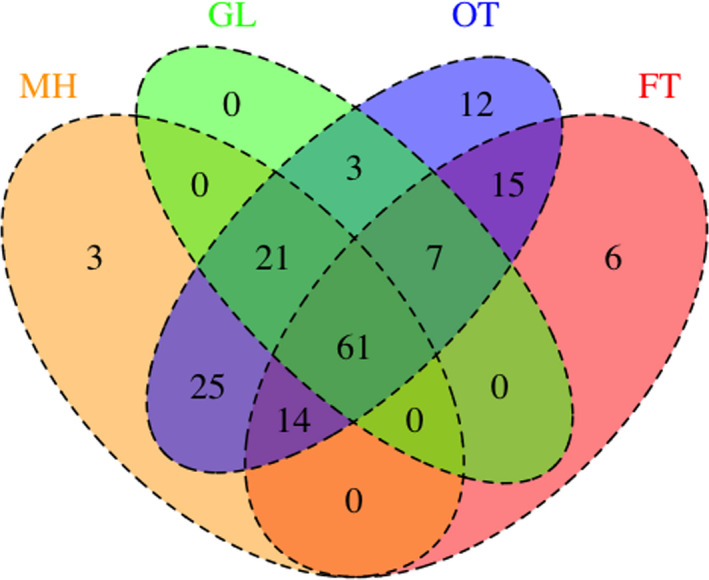
The Venn diagram showing the sharing of insect pollinators between four different habitats of Shivapuri‐Nagarjun National Park. Habitat: FT‐ Forest trail with canopy, GL‐ Grassland, MH‐ Managed habitat, and OT‐ Open trail of the forest without canopy. Color‐coding: FT (Pink), GL (green), MH (Skin), OT (Blue)

Among 167 species, the highest number of species was found in the Open trail (158), followed by the Managed habitat (124), Forest trail (103), and Grassland (92). Sixty‐one species were common in all of the habitats while the number of species exclusively found in one of the habitats was 12 for OT, 6 for FT, and 3 for MH (Figure 5). While comparing two types of habitats, the high number of habitats sharing of species was found between Open trail and Managed habitat (121) followed by Open trail and Forest trail (97), Grassland and Open trail (92), and Grassland and Managed habitat (82).

Whittaker Beta diversity showed the high species turnover between Forest trail and Managed habitat, whereas accounted for low species turnover between Managed habitat and Open trail (Table 3). Overall, Shannon Diversity of pollinators in SNNP (Gamma diversity) was 4.683 with 167 species (Supplementary 1).

**TABLE 3:** Whittaker Beta Diversity among four different habitats of Shivapuri‐Nagarjun National Park, Nepal

	FT	GL	MH	OT
FT	0	0.30	0.34	0.26
GL	0.30	0	0.24	0.26
MH	0.34	0.24	0	0.14
OT	0.26	0.26	0.14	0

Note

Habitat: FT–Forest trail with Canopy, GL–Grassland, MH–Managed habitat, and OT–the open trail of the forest without canopy.

### 3.4 Community composition of insect pollinators

#### 3.4.1 NMDS

**FIGURE 6:**
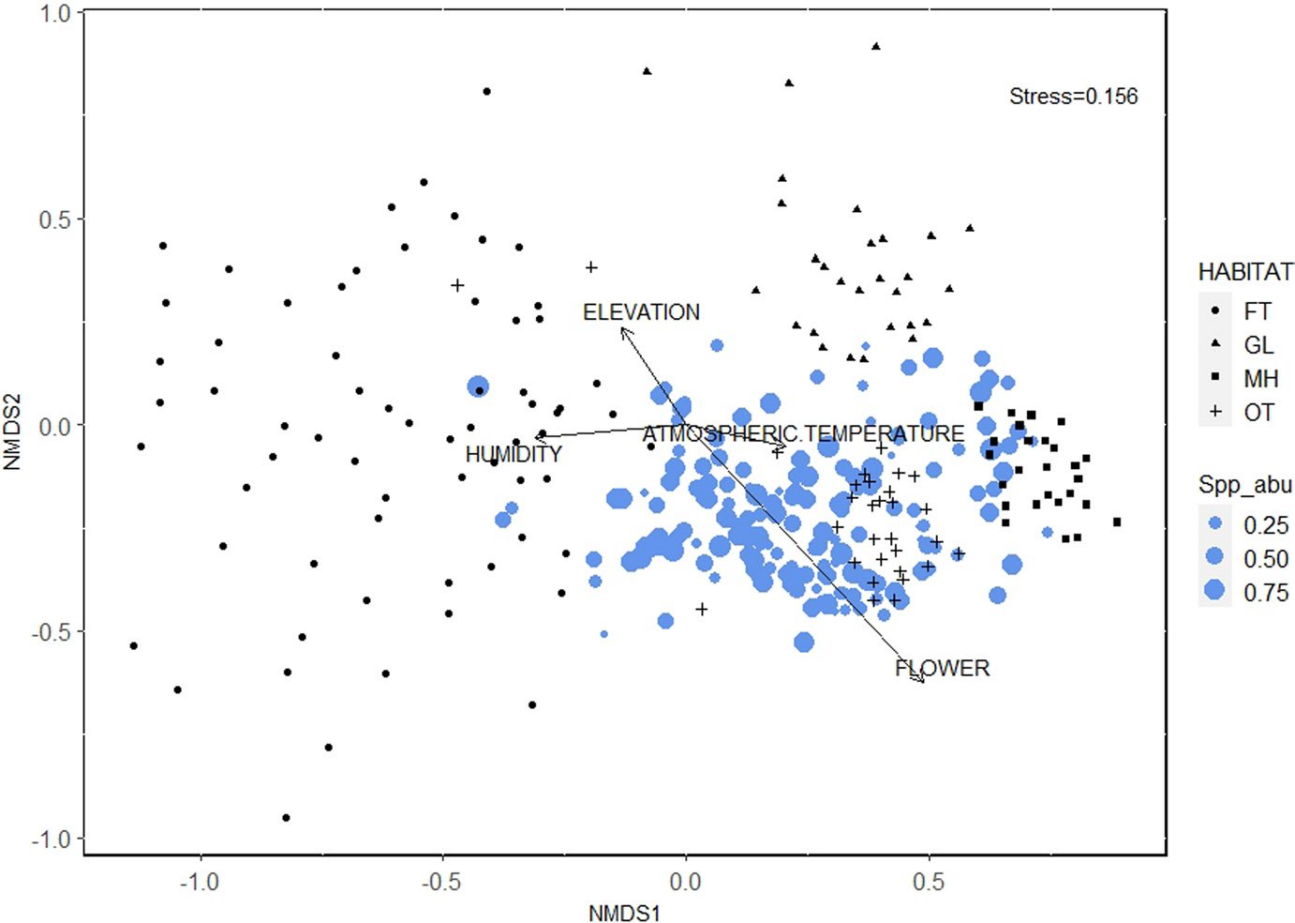
NMDS showing the influence of environmental variables on the community composition of insect pollinators in four different habitats of Shivapuri‐Nagarjun National Park. Habitat: FT‐ Forest trail with canopy, GL‐ Grassland, MH‐ Managed habitat, and OT‐ Open trail of the forest without canopy

The distance matrix of Bray–Curtis dissimilarity was calculated to plot Non‐metric Multidimensional Scaling (NMDS) to analyze insect pollinator's communities in four studied habitats (Forest trail, Grassland, Managed habitat, and Open trail). A stress value of 0.15 cleared convergence of NMDS ordination. Vector fitting of environmental variables showed that elevation, humidity, atmospheric temperature, and presence of flower resources have a significant association with pollinating insects (Table 4; Figure 6). Flower possessed significantly high NMDS1 score value of 0.62 (*p* < .001, *R* ^2^ = .63). Elevation and humidity were significantly negative with NMDS1 score value −0.51 (*p* = .009, *R* ^2^ = .07) and −1.00 (*p* < .001, *R* ^2^ = .10), respectively (Table 4). The highest compositional abundances of species were found associated with the flower resources which was at lower elevation (Positive end of NMDS1). Similarly, high species compositional abundance was seen in the Managed habitat and Open trail (Positive end of NMDS1) which were again at low elevation.

**TABLE 4:** NMDS scores on the axes of NMDS1 and NMDS2, significance values and coefficients of determination for the assessed environmental variables

	NMDS1	NMDS2	*R* ^2^	Pr(>*r*)
Elevation	–0.51	0.86	.07	.009^**^
Humidity	–1.00	–0.09	.10	.001^***^
Atmospheric Temperature	0.97	–0.25	.05	.036^*^
Flower	0.62	–0.79	.63	.001^***^

Note

Significant codes: 0’*** ‘ 0.001 ‘** ‘ 0.01 ‘* ‘ 0.05 ‘.’ 0.1 ‘ ‘ 1.

Permutation: free.

Number of permutations: 999.

Pollinators composition in four habitats is significantly different (Adonis, *p* < .001) with a significantly strong strength (ANOSIM, *R* = 0.62, *p* < .001). SIMPER analysis showed that *Xylocopa tenuiscapa* (0.70), *Danaus genutia* (0.70), and *Pelopidas agna* (0.69) were three species for different compositions between Forest trail and Managed habitat. Similarly, *Ceratina dentipes* (0.70), *Xylocopa tenuiscapa* (0.69), and *Graphium sarpedon* (0.69) were three species for the difference in composition between Forest trail and Open trail. Furthermore, *Syritta orientalis* (0.70), *Eristalis himalayensis* (0.70), and *Lasioglossum albipes* (0.69) were three species to contribute to the difference in the composition between Forest trail and Grassland. In Managed habitat and Open trail, *Andrena kathmanduensis* (0.70), *Neptis hylas* (0.70), and *Andrena gorkhana* (0.69) were three species to alter the pollinator's composition, while *Elaphropoda impatiens* (0.70), *Lethe verma* (0.70), and *Precis iphita* (0.69) in Managed habitat and Grassland. In the Open trail and Grassland, *Eristalinus taeniops* (0.70), *Pieris brassica* (0.70), and *Graphium sarpedon* (0.68) were three species indifferent in the composition.

#### 3.4.2 RDA

**FIGURE 7:**
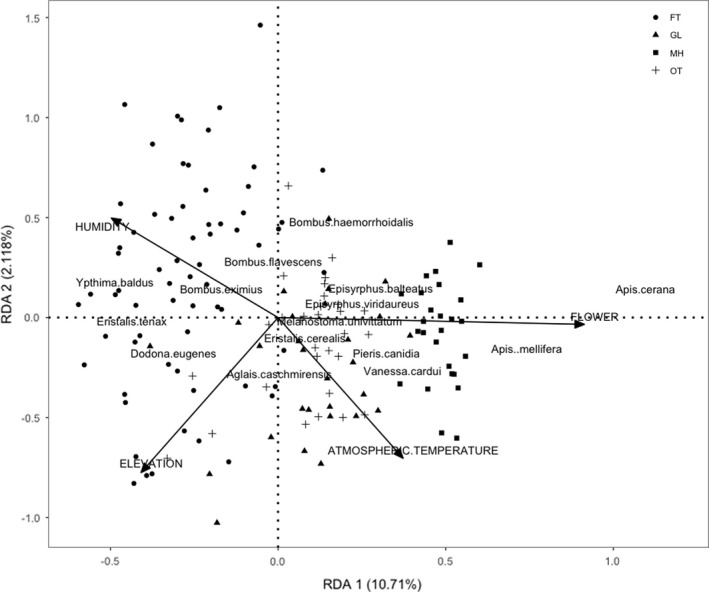
Redundancy Analysis (RDA) ‐ biplot showing the correlation between insect pollinator species and the environmental variables. Only top 15 most abundance (five from each group; bee, butterfly, and hoverfly) of the total species are shown on the plot. Habitat: FT‐Forest trail with canopy, GL‐ Grassland, MH‐Managed habitat, and OT‐Open trail of the forest without canopy

The correlation of environmental variables and the top 15 most abundance pollinator's community was shown by RDA ordination method (Figure 7). *Apis mellifera* or *A*. *cerana* use an active recruitment system (Proesmans et al., 2019; Winfree et al., 2007), which may result in an overabundance at certain spots that do not reflect the actual density of the species and vice versa. Thus, the analyses with *Apis* spp. included and excluded were carried out to estimate the effect of *Apis* spp. on the parameters assessed. The results showed that there were no such differences (Table 5). Hence, the *Apis* spp. were also included in the analysis. Two canonical axes of RDA analysis explained the variance by 12.83% in the pollinator–environmental interaction where the first axis and second axis accounted for 10.71% and 2.12% of the variance, respectively. Environmental factors: elevation and abundance of flower resources were found to be significantly associated with top 15 most abundant pollinators (*p* < .05), whereas humidity and atmospheric temperature did not have a significant impact on them. Managed habitat type represented the higher abundance of flowers associated with the high number of *Apis cerana* and *Apis mellifera*. On the other hand, *Pieris canidia* was found associated with both Open trail and Grassland and *Vanessa cardui* in a Managed habitat. *Bombus eximius*, *Bombus flavescens*, *Eristalis tenax*, and *Ypthima baldus* were more associated with the Forest trail which was a relatively humid area. Likewise, *Melanostoma univittatum* and *Episyrphus viridaureus* were associated with the Open trail, whereas *Episyrphus balteatus* was close to Grassland and Managed habitat.

**TABLE 5:** RDA presents correlation of environmental variables with the top 15 most abundance insect pollinators with analysis including *Apis* spp. (In.) and excluding *Apis* spp. (Ex.)

	df	Variance		*F*		Pr(>*F*)	
		In.	Ex.	In.	Ex.	In.	Ex.
Elevation	1	0.006	0.004	3.248	2.052	0.002**	0.025*
Humidity	1	0.003	0.004	1.588	1.700	0.099	0.070.
Atmospheric Temperature	1	0.003	0.003	1.238	1.533	0.250	0.107
Flower	1	0.021	0.011	10.651	5.568	0.001^***^	0.001^***^
residue	145	0.288	0.297				

Note

Significant codes: 0’*** ‘ 0.001 ‘** ‘ 0.01 ‘* ‘ 0.05 ‘.’ 0.1 ‘ ‘ 1’.

## 4 DISCUSSION

### 4.1 Abundance and species richness of pollinators in different habitats

Our present findings show that there are differences in the abundance and species richness of pollinators in the habitat types of SNNP. Such influence of habitat types in pollinators has also been reported in a tropical megacity, Bangkok, Thailand (Stewart et al., 2018). Our results indicate that the Open trail of the forest possesses the highest abundance and species richness of pollinating insects. The result reflects a direct relation of insects with the presence of flower resources. The Open trails of forest harbor a comparatively thick layer of herbs and shrubs with varieties of wildflowers that suffice pollinators with nectar and pollen sources. The availability of food for insect visitors is measured by flower resources (Baldock et al., 2015; Sjödin et al., 2008) which is one of the consistent and important variables (Ahrne et al., 2009; Hülsmann et al., 2015; Stewart et al., 2018) observed globally. The quality and quantity of nectar and pollen play a major role in the presence of pollinators (Hicks et al., 2016). Trails of open forest were densely proliferated and covered by *Ageratina adenophora* along with other wild herbs providing a good resource platform especially for hoverflies and bees. Furthermore, weeds like *Ranunculus repens* and *Trifolium repens* were comparatively more abundant in the Open trail. These flowers were preferred by both bees and butterflies (Chaguthi & Dyola, 2018; Hicks et al., 2016). These plants provide nectar and pollen for the insects (Erbar & Leins, 2013; Masters & Emery, 2015). Adult hoverflies require high energy for hovering flight that could be obtained from the local landscape with abundant flowers (Haslett, 1989; Meyer et al., 2009; Proesmans et al., 2019). In contrast, the presence of low flower resources also accounts for the decline in pollinator abundance and species richness in Forest trail with canopy. Canopy cover increases shade in the understory herb and shrub of the forest lowering flower blooms and limiting pollinator's movement (Proesmans et al., 2019). Sampled areas of the Managed habitat in SNNP were open, inhabited, and disturbed by some human activities. Some previous study also shows that the diversity of butterfly is negatively influenced by this factor of human disturbances (Kambach et al., 2013).

### 4.2 Diversity and distribution pattern of insect pollinators

Furthermore, our result depicts that Open trail is more diverse followed by Managed habitat. There is low species turnover between these two habitats. This could again be explained by the influence of the local environment between habitats. These two habitat types are open along with the presence of thicker beds of flower resources. Most of the pollinators show a strong preference for the structurally different land‐use type that add variety in resources they required (Bates et al., 2011; Matteson & Langellotto, 2010). However, flower resources are comparatively less in the Forest trail and Grassland, which could be the probable reason for less diverse pollinators and similar evenness of pollinators between these two habitats.

The distribution pattern of pollinators is varied among different species in the present study. Among all pollinators, 61 species were common in all the studied habitats. This result reflects the different needs of pollinators for different activities. There is a heterogeneous habitat choice of wild bees and a wider temporal range of activities of flies (Willcox et al., 2019). Many bee groups require different habitats for nesting and foraging (Franzén et al., 2009) and also different spatial foraging distances made by bees like *Bombus* spp. and *Xylocopa* spp. (Greenleaf et al., 2007). This could be a probable reason for the presence of some bees group in all habitats. Likewise, hoverflies feed on varieties of food resources in different stages of their life. For instance, the larva of some hoverfly develops in a close canopy while adults of the same fly hover in open flower‐rich biotopes (Gittings et al., 2006). Furthermore, the abundance of hoverflies increases with proximity to the forest (Moquet et al., 2018) and some flies are confined to only one kind of habitat like *Volucella trifasciata* and *Baccha maculata* which were recorded from forest habitats only. The latter species usually prefer the shady area of forest (Coe, 1964; Hassan et al., 2019). Butterflies too, show a different kind of habitats preference in their life cycles (Janz, 2005). Females dwell in grassland for oviposition while forage in flower‐rich habitats (Evans et al., 2020).

### 4.3 The community composition of insect pollinators

#### 4.3.1 NMDS

There is an influence of environmental variables in the community composition of pollinators. In our findings, the measured environmental variables (elevation, humidity, air temperature, and flower resources) are found to have a significant association with the pollinator's community. Species abundance of pollinators was concentrated in the Managed habitat and Open trail which were open to the high amount of solar exposure. Activities of the insect pollinators are highly influenced by such sun exposures (McKinney & Goodell, 2010; Sydenham et al., 2016). Similarly, flower resources were the next positive influencing factor for insect pollinators. The mixture of wildflowers could be a good attraction for all of these pollinators. A similar kind of relation of a different group was reported in the previous study (Carreck & Williams, 2002).

The complex environmental gradient of high altitude affects the abundance of different taxa of pollinators (Warren et al., 1988). As expected, with the elevation, the abundance of pollinators declined. The reason could be either decreasing of bee and fly attracted flowers or their limited number in higher altitude (Arnold et al., 2009). The plant communities at the high altitude limit the vascular plant and thus, availability of insect pollinators (Mani, 1962). The atmospheric temperature and abundance of flower resources have a negative correlation with elevation. Temperature and humidity (two oppositely related variables) are important factors for defining the plant composition at higher altitudes (Subedi et al., 2020) and hence alter pollinator's abundance. Only a few species of hoverflies that do not have specific food choices (Kearns, 1992) and big size bees, for instance, *Bombus* spp. that can thermoregulate and forage at low temperature, were only found in the highest altitude of our study area.

#### 4.3.2 RDA

The species of pollinators are differently associated with measured environmental variables in this study. *Apis cerana* and *A*. *mellifera* were mainly determined by the abundance of flower resources of the Managed habitat, while *Bombus eximius* and *B*. *flavescens* were associated with humid forest. The explanation for the *Apis* spp. could be their preference on floral resources near the nesting area like small size bees (Gathmann & Tscharntke, 2002; Vulliamy et al., 2006) so that they could gather maximum nectar to support the large bee colony (Potts et al., 2003). Large body‐sized bees like *Bombus* spp. could travel away from the nesting area for foraging (Greenleaf et al., 2007) and humid areas to avoid hotter day temperature (Willmer, 1983). *Aglais caschmirensis* is the most abundant and frequently seen butterfly in all kinds of habitats (Irungbam et al., 2017). This could be the reason for its presence in the forest as well as Managed habitat in our study area. Similarly, the association of *Episyrphus balteatus* in the Managed habitat indicates the preference for flowers of vegetables such as coriander, buckwheat (Pinheiro et al., 2015) as their host plant.

## 5 CONCLUSIONS

We studied the diversity, distribution, and community structure of insect pollinators in different habitats of SNNP, Nepal. Overall, habitats, humidity, atmospheric temperature, abundance of flower resources, and elevation played a significant role in the diversity, distribution, and community structure of pollinating insects. Insect pollinators were strongly associated with flower resources which were highly recorded in an Open trail. Shannon Index and evenness were high in an Open trail and similar evenness was found in the Forest trail and Grassland. Conservation of plant diversity in the walking trails of SNNP is important to conserve the community structure of insect pollinators.

## CONFLICT OF INTEREST

The authors declare that they have no conflict of interest.

## AUTHOR CONTRIBUTIONS

**Urmila Dyola:** Conceptualization (lead); Data curation (lead); Formal analysis (lead); Funding acquisition (lead); Investigation (lead); Writing – original draft (lead); Writing – review & editing (lead). **Chitra Bahadur Baniya:** Conceptualization (equal); Formal analysis (equal); Investigation (supporting); Supervision (equal); Writing – review & editing (supporting). **Pushpa Raj Acharya:** Conceptualization (equal); Supervision (equal); Writing – review & editing (supporting). **Pradip Subedi:** Data curation (supporting); Formal analysis (supporting); Investigation (supporting). **Anjeela Pandey:** Data curation (supporting); Investigation (supporting). **Kumar Sapkota:** Conceptualization (lead); Investigation (supporting); Supervision (lead); Writing – review & editing (supporting).

## Supporting information

Supplementary Material 1

Supplementary Material 2

Supplementary Material 3

## Data Availability

The supplementary data associated with this manuscript are available at Dryad: https://doi.org/10.5061/dryad.9zw3r22g1.

## References

[ece38653-bib-0001] Adedoja, O. , Kehinde, T. , & Samways, M. J. (2020). Asynchrony among insect pollinator groups and flowering plants with elevation. Scientific Reports, 10(1), 1–12. 10.1038/s41598-020-70055-5 32764658 PMC7411018

[ece38653-bib-0002] Ahrne, K. , Bengtsson, J. , & Elmqvist, T. (2009). Bumble bees (Bombus spp) along a gradient of increasing urbanization. PLoS One, 4(5), e5574. 10.1371/journal.pone.0005574 19440367 PMC2679196

[ece38653-bib-0003] Amatya, D. (1993). Forest vegetation analysis. Development Project, GCP/NEP/048/NOR. Kathmandu: His Majesty's Government of Nepal and Food and Agriculture Organisation of the United Nations and possible ecological implications. Brazilian Journal of Biology, 64, 563–568.

[ece38653-bib-0004] Arnold, S. E. , Le Comber, C. S. , & Chittka, L. (2009). Flower color phenology in European grassland and woodland habitats, through the eyes of pollinators. Israel Journal of Plant Sciences, 57(3), 211–230. 10.1560/IJPS.57.3.211

[ece38653-bib-0005] Aslam, A. , Rafi, M. A. , & Zia, A. (2017). Non‐Apis bees of family Apidae (Hymenoptera) from Potohar region of Pakistan. Journal of Entomology and Zoology Studies, 5(2), 6–12.

[ece38653-bib-0006] Baldock, K. C. R. , Goddard, M. A. , Hicks, D. M. , Kunin, W. E. , Mitschunas, N. , Osgathorpe, L. M. , Potts, S. G. , Robertson, K. M. , Scott, A. V. , Stone, G. N. , Vaughan, I. P. , & Memmott, J. (2015). Where is the UK's pollinator biodiversity? The importance of urban areas for flower–visiting insects. Proceedings of the Royal Society B: Biological Sciences, 282(1803), 20142849. 10.1098/rspb.2014.2849 PMC4345454 25673686

[ece38653-bib-0007] Bashir, M. A. , Saeed, S. , Sajjad, A. , Khan, K. A. , Ghramh, H. A. , Shehzad, M. A. , Mubarak, H. , Mirza, N. , Mahpara, S. , Rehmani, M. I. A. , & Ansari, M. J. (2019). Insect pollinator diversity in four forested ecosystems of southern Punjab, Pakistan. Saudi Journal of Biological Sciences, 26(7), 1835–1842. 10.1016/j.sjbs.2018.02.007 31762665 PMC6864159

[ece38653-bib-0008] Bates, A. J. , Sadler, J. P. , Fairbrass, A. J. , Falk, S. J. , Hale, J. D. , & Matthews, T. J. (2011). Changing bee and hoverfly pollinator assemblages along an urban–rural gradient. PLoS One, 6(8), e23459. 10.1371/journal.pone.0023459 21858128 PMC3155562

[ece38653-bib-0009] Bingham, C. T. (1897). The Fauna of British India, Including Ceylon and Burma. Hymenoptera, 1. Wasps and Bees. Taylor & Francis.

[ece38653-bib-0010] Bodlah, I. , Amjad, M. , Bodlah, M. A. , & Qayyum, A. (2016). First record of two genera of Anthophorini and one genus of Melectini (Apinae: Apidae: Hymenoptera) from Pothwar Punjab, Pakistan. Journal of Entomology and Zoology Studies, 4, 1031–1035.

[ece38653-bib-0011] Brunetti, E. (1923). Pipunculidae, Syrphidae, Conopidae, Oestridae. In A. E. Shipley (Ed.), The Fauna of British India, Including Ceylon and Burma. (Diptera. Vol. 3, xi +424 pp., 6 pis). Taylor and Francis.

[ece38653-bib-0012] Cameron, S. A. , Lozier, J. D. , Strange, J. P. , Koch, J. B. , Cordes, N. , Solter, L. F. , & Griswold, T. L. (2011). Patterns of widespread decline in North American bumble bees. Proceedings of the National Academy of Sciences, 108(2), 662–667. 10.1073/pnas.1014743108 PMC3021065 21199943

[ece38653-bib-0013] Carreck, N. L. , & Williams, I. H. (2002). Food for insect pollinators on farmland: Insect visits to flowers of annual seed mixtures. Journal of Insect Conservation, 6(1), 13–23.

[ece38653-bib-0014] Carvalheiro, L. G. , Kunin, W. E. , Keil, P. , Aguirre‐Gutiérrez, J. , Ellis, W. N. , Fox, R. , Groom, Q. , Hennekens, S. , Van Landuyt, W. , Maes, D. , & Van de Meutter, F. (2013). Species richness declines and biotic homogenisation have slowed down for NW‐European pollinators and plants. Ecology Letters, 16(7), 870–878. 10.1111/ele.12121 23692632 PMC3738924

[ece38653-bib-0015] Chaguthi, G. , & Dyola, U. (2018). Insect visitors of white clover (*Trifolium repens* L) and their relation with environmental variables in the premises of bhaktapur multiple campus, Nepal. Journal of Institute of Science and Technology, 22(2), 86–91. 10.3126/jist.v22i2.19598

[ece38653-bib-0016] Clarke, K. R. , & Gorley, R. N. (2001). Primer V5 (Plymouth routines in multivariate ecological research): User manual/tutorial. Primer–e.

[ece38653-bib-0017] Claussen, C. J. , & Weipert, J. (2003). Zur Schwebfliegenfauna Nepals (Insecta: Diptera: Syrphidae) unter besonderer Berücksichtigung Westnepals. In Biodiversität und Naturausstattung im Himalaya. Verein der Freunde und Förderer des Naturkundemuseums Erfurt ev, Erfurt (pp. 343–380).

[ece38653-bib-0018] Coe, R. L. (1964). Diptera from Nepal. Bulletin of the British Museum of Natural History, Entomology, 15, 255–290.

[ece38653-bib-0019] Corbet, S. A. , Williams, I. H. , & Osborne, J. L. (1991). Bees and the pollination of crops and wild flowers in the European Community. Bee World, 72(2), 47–59. 10.1080/0005772X.1991.11099079

[ece38653-bib-0020] De Groot, R. S. , Alkemade, R. , Braat, L. , Hein, L. , & Willemen, L. (2010). Challenges in integrating the concept of ecosystem services and values in landscape planning, management and decision making. Ecological Complexity, 7(3), 260–272. 10.1016/j.ecocom.2009.10.006

[ece38653-bib-0021] Dirzo, R. , Young, H. S. , Galetti, M. , Ceballos, G. , Isaac, N. J. , & Collen, B. (2014). Defaunation in the anthropocene. Science, 345(6195), 401–406. 10.1126/science.1251817 25061202

[ece38653-bib-0022] Erbar, C. , & Leins, P. (2013). Nectar production in the pollen flower of *Anemone nemorosa* in comparison with other Ranunculaceae and Magnolia (Magnoliaceae). Organisms Diversity and Evolution, 13(3), 287–300. 10.1007/s13127-013-0131-9

[ece38653-bib-0023] Evans, L. C. , Sibly, R. M. , Thorbek, P. , Sims, I. , Oliver, T. H. , & Walters, R. J. (2020). The importance of including habitat–specific behaviour in models of butterfly movement. Oecologia, 193(2), 249–259. 10.1007/s00442-020-04638-4 32253493 PMC7320960

[ece38653-bib-0024] Everaars, J. , Strohbach, M. W. , Gruber, B. , & Dormann, C. F. (2011). Microsite conditions dominate habitat selection of the red mason bee (Osmia bicornis, Hymenoptera: Megachilidae) in an urban environment: A case study from Leipzig. Germany. Landscape and Urban Planning, 103(1), 15–23. 10.1016/j.landurbplan.2011.05.008

[ece38653-bib-0025] Fitzpatrick, Ú. , Murray, T. E. , Paxton, R. J. , Breen, J. , Cotton, D. , Santorum, V. , & Brown, M. J. (2007). Rarity and decline in bumblebees–a test of causes and correlates in the Irish fauna. Biological Conservation, 136(2), 185–194. 10.1016/j.biocon.2006.11.012

[ece38653-bib-0026] Franzén, M. , Larsson, M. , & Nilsson, S. G. (2009). Small local population sizes and high habitat patch fidelity in a specialised solitary bee. Journal of Insect Conservation, 13(1), 89–95. 10.1007/s10841-007-9123-4

[ece38653-bib-0027] Fussell, M. , & Corbet, S. A. (1992). Flower usage by bumble–bees: A basis for forage plant management. Journal of Applied Ecology, 451–465. 10.2307/2404513

[ece38653-bib-0028] Gathmann, A. , & Tscharntke, T. (2002). Foraging ranges of solitary bees. Journal of Animal Ecology, 71(5), 757–764. 10.1046/j.1365-2656.2002.00641.x

[ece38653-bib-0029] Ghorpadé, K. (1994). Diagnostic keys to new and known genera and species of Indian subcontinent Syrphini (Diptera: Syrphidae). Colemania: Insect Biosystematics, 3, 1–15.

[ece38653-bib-0030] Ghorpadé, K. (2019). Hover‐flies (Diptera: Syrphidae) recorded from “Dravidia,” or Central and Peninsular India and Sri Lanka. An Annotated Checklist and Bibliography. In Ramani, S. , Mohanraj, P. , & Yeshwanth, H. (Eds.), Indian insects: Diversity and science (Vol. 472, pp. 325–388). CRC Press. 10.1201/9780429061400-18

[ece38653-bib-0031] Gilbert, L. E. (1972). Pollen feeding and reproductive biology of Heliconius butterflies. Proceedings of the National Academy of Sciences, 69(6), 1403–1407. 10.1073/pnas.69.6.1403 PMC426712 16591992

[ece38653-bib-0032] Gill, R. J. , Baldock, K. C. , Brown, M. J. , Cresswell, J. E. , Dicks, L. V. , Fountain, M. T. , Garratt, M. P. , Gough, L. A. , Heard, M. S. , Holland, J. M. , & Ollerton, J. (2016). Protecting an ecosystem service: Approaches to understanding and mitigating threats to wild insect pollinators. Advances in Ecological Research, 54, 135–206. 10.1016/bs.aecr.2015.10.007

[ece38653-bib-0033] Gittings, T. , O’Halloran, J. , Kelly, T. , & Giller, P. S. (2006). The contribution of open spaces to the maintenance of hoverfly (Diptera, Syrphidae) biodiversity in Irish plantation forests. Forest Ecology and Management, 237(1–3), 290–300. 10.1016/j.foreco.2006.09.052

[ece38653-bib-0034] Greenleaf, S. S. , Williams, N. M. , Winfree, R. , & Kremen, C. (2007). Bee foraging ranges and their relationship to body size. Oecologia, 153(3), 589–596. 10.1007/s00442-007-0752-9 17483965

[ece38653-bib-0035] Hammer, Ø. , Harper, D. A. , & Ryan, P. D. (2001). PAST: Paleontological statistics software package for education and data analysis. Palaeontologia Electronica, 4(1), 9.

[ece38653-bib-0036] Haslett, J. R. (1989). Adult feeding by holometabolous insects: Pollen and nectar as complementary nutrient sources for Rhingia campestris (Diptera: Syrphidae). Oecologia, 81(3), 361–363. 10.1007/BF00377084 28311189

[ece38653-bib-0037] Hassan, M. A. , Bodlah, I. , Ahmad, M. , Kayani, A. R. , & Mahmood, K. (2020). First record of the genus Graptomyza Wiedemann, 1830 (Diptera: Syrphidae) from Pakistan. The Journal of Animal and Plant Sciences, 30(2), 512–516.

[ece38653-bib-0038] Hassan, M. A. , Bodlah, I. , Aihetasham, A. , Bodlah, M. A. , & Hussain, K. (2019). First Record of Baccha maculata Walker, 1852 (Diptera: Syrphidae) from the Pothwar Punjab, Pakistan. Punjab University Journal of Zoology, 34(2), 133–135. 10.17582/journal.pujz/2019.34.2.133.135

[ece38653-bib-0039] Herrera, C. M. (1987). Components of pollinator" quality": Comparative analysis of a diverse insect assemblage. Oikos, 50, 79–90. 10.2307/3565403

[ece38653-bib-0040] Hicks, D. M. , Ouvrard, P. , Baldock, K. C. R. , Baude, M. , Goddard, M. A. , Kunin, W. E. , Mitschunas, N. , Memmott, J. , Morse, H. , Nikolitsi, M. , Osgathorpe, L. M. , Potts, S. G. , Robertson, K. M. , Scott, A. V. , Sinclair, F. , Westbury, D. B. , & Stone, G. N. (2016). Food for pollinators: Quantifying the nectar and pollen resources of urban flower meadows. PLoS One, 11(6), e0158117. 10.1371/journal.pone.0158177 27341588 PMC4920406

[ece38653-bib-0041] Hudewenz, A. , Klein, A. M. , Scherber, C. , Stanke, L. , Tscharntke, T. , Vogel, A. , Weigelt, A. , Weisser, W. W. , & Ebeling, A. (2012). Herbivore and pollinator responses to grassland management intensity along experimental changes in plant species richness. Biological Conservation, 150(1), 42–52. 10.1016/j.biocon.2012.02.024

[ece38653-bib-0042] Hülsmann, M. , von Wehrden, H. , Klein, A. M. , & Leonhardt, S. D. (2015). Plant diversity and composition compensate for negative effects of urbanization on foraging bumble bees. Apidologie, 46(6), 760–770. 10.1007/s13592-015-0366-x

[ece38653-bib-0043] Irungbam, J. S. , Huidrom, H. , & Soibam, B. S. (2017). Range extension of the Indian Tortoiseshell Aglais caschmirensis aesis (Fruhstorfer, 1912) (Lepidoptera: Nymphalidae) into the hills of Manipur, India. Journal of Threatened Taxa, 9(10), 10860–10864. 10.11609/jott.2983.9.10.10860-10864

[ece38653-bib-0044] Janz, N. (2005). The relationship between habitat selection and preference for adult and larval food resources in the polyphagous butterfly Vanessa cardui (Lepidoptera: Nymphalidae). Journal of Insect Behavior, 18(6), 767–780. 10.1007/s10905-005-8739-z

[ece38653-bib-0045] Jauker, F. , Diekoetter, T. , Schwarzbach, F. , & Wolters, V. (2009). Pollinator dispersal in an agricultural matrix: Opposing responses of wild bees and hoverflies to landscape structure and distance from main habitat. Landscape Ecology, 24(4), 547–555. 10.1007/s10980-009-9331-2

[ece38653-bib-0046] Jennersten, O. (1984). Flower visitation and pollination efficiency of some North European butterflies. Oecologia, 63(1), 80–89. 10.1007/BF00379789 28311170

[ece38653-bib-0047] Kambach, S. , Guerra, F. , Beck, S. G. , Hensen, I. , & Schleuning, M. (2013). Human–induced disturbance alters pollinator communities in tropical mountain forests. Diversity, 5(1), 1–14. 10.3390/d5010001

[ece38653-bib-0048] Kearns, C. A. (1992). Anthophilous fly distribution across an elevation gradient. American Midland Naturalist, 127, 172–182. 10.2307/2426332

[ece38653-bib-0049] Kearns, C. A. , Inouye, D. W. , & Waser, N. M. (1998). Endangered mutualisms: the conservation of plant–pollinator interactions. Annual Review of Ecology and Systematics, 29(1), 83–112. 10.1146/annurev.ecolsys.29.1.83

[ece38653-bib-0050] Kevan, P. G. , Clark, E. A. , & Thomas, V. G. (1990). Insect pollinators and sustainable agriculture. American Journal of Alternative Agriculture, 5(1), 13–22. 10.1017/S0889189300003179

[ece38653-bib-0051] Kovács‐Hostyánszki, A. , Espíndola, A. , Vanbergen, A. J. , Settele, J. , Kremen, C. , & Dicks, L. V. (2017). Ecological intensification to mitigate impacts of conventional intensive land use on pollinators and pollination. Ecology Letters, 20(5), 673–689. 10.1111/ele.12762 28346980 PMC6849539

[ece38653-bib-0052] Kremen, C. (2008). Crop pollination services from wild bees. In Bee pollination in agricultural ecosystems (pp. 10–26).

[ece38653-bib-0053] Kühsel, S. , & Blüthgen, N. (2015). High diversity stabilizes the thermal resilience of pollinator communities in intensively managed grasslands. Nature Communications, 6(1), 1–10. 10.1038/ncomms8989 PMC4918356 26258282

[ece38653-bib-0054] Kumari, P. , Kumar, N. R. , Sidhu, A. K. , & Chandra, K. (2018). Taxonomical and behavioural studies on Megachile conjuncta (Fabricius) (Hymenoptera: Megachilidae: Cressoniella).

[ece38653-bib-0055] Larson, B. M. H. , Kevan, P. G. , & Inouye, D. W. (2001). Flies and flowers: Taxonomic diversity of anthophiles and pollinators. The Canadian Entomologist, 133(4), 439–465. 10.4039/Ent133439-4

[ece38653-bib-0056] LeBuhn, G. , Griswold, T. , Minckley, R. , Droege, S. , Roulston, T. A. , Cane, J. , Parker, F. , Buchmann, S. , Tepedino, V. , Williams, N. , & Kremen, C. (2003). A standardized method for monitoring bee populations–the bee inventory (BI) plot. Accessed, 16, 15.

[ece38653-bib-0057] Lepš, J. , & Šmilauer, P. (2003). Multivariate analysis of ecological data using CANOCO. Cambridge University Press.

[ece38653-bib-0059] Losey, J. E. , & Vaughan, M. (2006). The economic value of ecological services provided by insects. BioScience, 56(4), 311–323. 10.1641/0006-3568(2006)56[311:TEVOES]2.0.CO;2

[ece38653-bib-0060] Mani, M. S. (1962). Introduction to high altitude entomology insect life above the timber–line in the North–west Himalaya (Vol. 31, pp. 48).

[ece38653-bib-0061] Masters, J. A. , & Emery, S. M. (2015). The showy invasive plant Ranunculus ficaria facilitates pollinator activity, pollen deposition, but not always seed production for two native spring ephemeral plants. Biological Invasions, 17(8), 2329–2337. 10.1007/s10530-015-0878-3

[ece38653-bib-0062] Matteson, K. C. , & Langellotto, G. A. (2010). Determinates of inner city butterfly and bee species richness. Urban Ecosystems, 13(3), 333–347. 10.1007/s11252-010-0122-y

[ece38653-bib-0063] McKinney, A. M. , & Goodell, K. (2010). Shading by invasive shrub reduces seed production and pollinator services in a native herb. Biological Invasions, 12(8), 2751–2763. 10.1007/s10530-009-9680-4

[ece38653-bib-0064] Meyer, B. , Jauker, F. , & Steffan‐Dewenter, I. (2009). Contrasting resource–dependent responses of hoverfly richness and density to landscape structure. Basic and Applied Ecology, 10(2), 178–186. 10.1016/j.baae.2008.01.001

[ece38653-bib-0065] Moquet, L. , Laurent, E. , Bacchetta, R. , & Jacquemart, A. L. (2018). Conservation of hoverflies (Diptera, Syrphidae) requires complementary resources at the landscape and local scales. Insect Conservation and Diversity, 11(1), 72–87. 10.1111/icad.12245 32336985 PMC7165621

[ece38653-bib-0066] Neumüller, U. , Burger, H. , Krausch, S. , Blüthgen, N. , & Ayasse, M. (2020). Interactions of local habitat type, landscape composition and flower availability moderate wild bee communities. Landscape Ecology, 35(10), 2209–2224. 10.1007/s10980-020-01096-4

[ece38653-bib-0067] Ngat, T. T. , Minh, N. P. , Lam, T. X. , Thi, N. , & Lien, P. (2017). Studies of the genus Thyreus panzer (Hymenoptera : Apidae : Apinae) with six new records from Vietnam. Biological Forum – an International Journal, 9(2), 227–236.

[ece38653-bib-0068] Nielsen, A. , Steffan‐Dewenter, I. , Westphal, C. , Messinger, O. , Potts, S. G. , Roberts, S. P. M. , Settele, J. , Szentgyörgyi, H. , Vaissière, B. E. , Vaitis, M. , Woyciechowski, M. , Bazos, I. , Biesmeijer, J. C. , Bommarco, R. , Kunin, W. E. , Tscheulin, T. , Lamborn, E. , & Petanidou, T. (2011). Assessing bee species richness in two Mediterranean communities: importance of habitat type and sampling techniques. Ecological Research, 26(5), 969–983. 10.1007/s11284-011-0852-1

[ece38653-bib-0069] Oksanen, J. , Blanchet, F. G. , Kindt, R. , Legendre, P. , Minchin, P. R. , O’Hara, R. B. , Simpson, G. L. , Solymos, P. , Stevens, M. H. H. , Wagner, H. , & Oksanen, M. J. (2013). Package ‘vegan’. Community Ecology Package, Version, 2(9), 1–295.

[ece38653-bib-0070] Oliver, T. , Roy, D. B. , Hill, J. K. , Brereton, T. , & Thomas, C. D. (2010). Heterogeneous landscapes promote population stability. Ecology Letters, 13(4), 473–484. 10.1111/j.1461-0248.2010.01441.x 20148927

[ece38653-bib-0071] Pellissier, L. , Pottier, J. , Vittoz, P. , Dubuis, A. , & Guisan, A. (2010). Spatial pattern of floral morphology: Possible insight into the effects of pollinators on plant distributions. Oikos, 119(11), 1805–1813. 10.1111/j.1600-0706.2010.18560.x

[ece38653-bib-0072] Pinheiro, L. A. , Torres, L. M. , Raimundo, J. , & Santos, S. A. (2015). Effects of pollen, sugars and honeydew on lifespan and nutrient levels of Episyrphus balteatus. BioControl, 60(1), 47–57. 10.1007/s10526-014-9621-8

[ece38653-bib-0073] Pollard, E. , & Yates, T. J. (1994). Monitoring butterflies for ecology and conservation: The British butterfly monitoring scheme. Springer Science and Business Media.

[ece38653-bib-0074] Potts, S. G. , Biesmeijer, J. C. , Kremen, C. , Neumann, P. , Schweiger, O. , & Kunin, W. E. (2010). Global pollinator declines: Trends, impacts and drivers. Trends in Ecology and Evolution, 25(6), 345–353. 10.1016/j.tree.2010.01.007 20188434

[ece38653-bib-0075] Potts, S. G. , Vulliamy, B. , Dafni, A. , Ne'eman, G. , & Willmer, P. (2003). Linking bees and flowers: How do floral communities structure pollinator communities? Ecology, 84(10), 2628–2642. 10.1890/02-0136

[ece38653-bib-0076] Proesmans, W. , Bonte, D. , Smagghe, G. , Meeus, I. , & Verheyen, K. (2019). Importance of forest fragments as pollinator habitat varies with season and guild. Basic and Applied Ecology, 34, 95–107. 10.1016/j.baae.2018.08.004

[ece38653-bib-0077] R Core Team (2021). R: A language and environment for statistical computing. R Foundation for Statistical Computing. https://www.R‐project.org/

[ece38653-bib-0078] Reemer, M. (2005). Saproxylic hoverflies benefit by modern forest management (Diptera: Syrphidae). Journal of Insect Conservation, 9(1), 49–59. 10.1007/s10841-004-6059-9

[ece38653-bib-0079] Rundlöf, M. , Nilsson, H. , & Smith, H. G. (2008). Interacting effects of farming practice and landscape context on bumble bees. Biological Conservation, 141(2), 417–426. 10.1016/j.biocon.2007.10.011

[ece38653-bib-0080] Sankararaman, H. , Daniel, J. A. , Manickavasagam, S. , & Pennards, G. (2020). First record of two interesting genera of hover flies (Diptera: Syrphidae) in South India. Journal of Insect Biodiversity, 14(2), 54–63. 10.12976/jib/2020.14.2.4

[ece38653-bib-0081] Senapathi, D. , Goddard, M. A. , Kunin, W. E. , & Baldock, K. C. (2017). Landscape impacts on pollinator communities in temperate systems: Evidence and knowledge gaps. Functional Ecology, 31(1), 26–37. 10.1111/1365-2435.12809

[ece38653-bib-0082] Sengupta, J. , Naskar, A. , Maity, A. , Hazra, S. , Sarkar, N. K. , & Banerjee, D. (2017). Hover flies (Diptera: Syrphidae) from Darjeeling Himalaya—A part of indo‐Burmese hotspot. Indian Journal of Entomology, 79(3), 336–353. 10.5958/0974-8172.2017.00065.7

[ece38653-bib-0083] Sharp, M. A. , Parks, D. R. , & Ehrlich, P. R. (1974). Plant resources and butterfly habitat selection. Ecology, 55(4), 870–875. 10.2307/1934423

[ece38653-bib-0084] Sjödin, N. E. , Bengtsson, J. , & Ekbom, B. (2008). The influence of grazing intensity and landscape composition on the diversity and abundance of flower‐visiting insects. Journal of Applied Ecology, 45(3), 763–772. 10.1111/j.1365-2664.2007.01443.x

[ece38653-bib-0085] Smith, C. (2011). Illustrated checklists of Nepal’s butterflies. Mujpuria Publication, Craftman Press.

[ece38653-bib-0086] SNNP (2017). Shivapuri Nagarjun National Park and buffer zone management plan Fiscal Year. Shivapuri Nagarjun National Park office.

[ece38653-bib-0087] Stanley, D. A. (2013). Pollinators and Pollination in Changing Agricultural Landscapes: Investigating the Impacts of Bioenergy Crops (Doctoral dissertation, Trinity College Dublin).

[ece38653-bib-0088] Stanley, D. A. , Gunning, D. , & Stout, J. C. (2013). Pollinators and pollination of oilseed rape crops (*Brassica napus* L.) in Ireland: ecological and economic incentives for pollinator conservation. Journal of Insect Conservation, 17(6), 1181–1189. 10.1007/s10841-013-9599-z

[ece38653-bib-0089] Stewart, A. B. , Sritongchuay, T. , Teartisup, P. , Kaewsomboon, S. , & Bumrungsri, S. (2018). Habitat and landscape factors influence pollinators in a tropical megacity, Bangkok. Thailand. Peerj, 6, e5335. 10.7717/peerj.5335 30042902 PMC6055598

[ece38653-bib-0090] Subedi, C. K. , Rokaya, M. B. , Münzbergová, Z. , Timsina, B. , Gurung, J. , Chettri, N. , Baniya, C. B. , Ghimire, S. K. , & Chaudhary, R. P. (2020). Vascular plant diversity along an elevational gradient in the Central Himalayas, western Nepal. Folia Geobotanica, 55(2), 127–140. 10.1007/s12224-020-09370-8

[ece38653-bib-0091] Sydenham, M. A. , Häusler, L. D. , Moe, S. R. , & Eldegard, K. (2016). Inter‐assemblage facilitation: The functional diversity of cavity‐producing beetles drives the size diversity of cavity‐nesting bees. Ecology and Evolution, 6(2), 412–425. 10.1002/ece3.1871 26843927 PMC4729264

[ece38653-bib-0092] Szigeti, V. , Kőrösi, Á. , Harnos, A. , Nagy, J. , & Kis, J. (2016). Comparing two methods for estimating floral resource availability for insect pollinators in semi‐natural habitats. Annales De La Société Entomologique De France (N.S.), 52(5), 289–299. 10.1080/00379271.2016.1261003

[ece38653-bib-0093] Tadauchi, O. , & Matsumura, T. (2007). The genus Andrena collected from Nepal (Hymenoptera, Andrenidae) with redescriptions of some types of Andrena described from North India. Esakia, 47, 1–20. 10.5109/8322

[ece38653-bib-0094] Thompson, F. C. , & Ghorpade, K. (1992). A new coffee aphid predator, with notes on other Oriental species of Paragus (Diptera: Syrphidae). Colemania, 5, 1–24.

[ece38653-bib-0095] Tiple, A. D. , Deshmukh, V. P. , & Dennis, R. L. (2005). Factors influencing nectar plant resource visits by butterflies on a university campus: Implications for conservation. Nota Lepidopterologica, 28(3/4), 213.

[ece38653-bib-0096] Van Swaay, C. C. , Cuttelod, A. , Collins, S. , Maes, D. , Munguira, M. L. , Šašić, M. , Settele, J. , Verovnik, R. , Verstrael, T. , Warren, M. , & Wiemers, M. (2010). European Red List of Butterflies. 10.2779/83897

[ece38653-bib-0097] Vanbergen, A. J. , & Initiative, T. I. P. (2013). Threats to an ecosystem service: pressures on pollinators. Frontiers in Ecology and the Environment, 11(5), 251–259. 10.1890/120126

[ece38653-bib-0098] Vulliamy, B. , G. Potts, S. , & G. Willmer, P. (2006). The effects of cattle grazing on plant‐pollinator communities in a fragmented Mediterranean landscape. Oikos, 114(3), 529–543. 10.1111/j.2006.0030-1299.14004.x

[ece38653-bib-0099] Warren, M. S. , Hill, J. K. , Thomas, J. A. , Asher, J. , Fox, R. , Huntley, B. , Roy, D. B. , Telfer, M. G. , Jeffcoate, S. , Harding, P. , Jeffcoate, G. , Willis, S. G. , Greatorex‐Davies, J. N. , Moss, D. , & Thomas, C. D. (2001). Rapid responses of British butterflies to opposing forces of climate and habitat change. Nature, 414(6859), 65–69. 10.1038/35102054 11689943

[ece38653-bib-0100] Warren, S. D. , Harper, K. T. , & Booth, G. M. (1988). Elevational distribution of insect pollinators. American Midland Naturalist, 325–330. 10.2307/2426004

[ece38653-bib-0101] Weibull, A. C. , Bengtsson, J. , & Nohlgren, E. (2000). Diversity of butterflies in the agricultural landscape: The role of farming system and landscape heterogeneity. Ecography, 23(6), 743–750. 10.1111/j.1600-0587.2000.tb00317.x

[ece38653-bib-0102] Westphal, C. , Bommarco, R. , Carré, G. , Lamborn, E. , Morison, N. , Petanidou, T. , Potts, S. G. , Roberts, S. P. M. , Szentgyörgyi, H. , Tscheulin, T. , Vaissière, B. E. , Woyciechowski, M. , Biesmeijer, J. C. , Kunin, W. E. , Settele, J. , & Steffan‐Dewenter, I. (2008). Measuring bee diversity in different European habitats and biogeographical regions. Ecological Monographs, 78(4), 653–671. 10.1890/07-1292.1

[ece38653-bib-0103] Wickham, H. , Chang, W. , & Wickham, M. H. (2016). Package ‘ggplot2’. Create Elegant Data Visualisations Using the Grammar of Graphics. Version, 2(1), 1–189.

[ece38653-bib-0104] Widhiono, I. , Sudiana, E. , & Sucianto, E. T. (2016). Insect pollinator diversity along a habitat quality gradient on Mount Slamet, Central Java, Indonesia. Biodiversitas Journal of Biological Diversity, 17(2). 10.13057/biodiv/d170250

[ece38653-bib-0105] Willcox, B. K. , Howlett, B. G. , Robson, A. J. , Cutting, B. , Evans, L. , Jesson, L. , Kirkland, L. , Jean‐Meyzonnier, M. , Potdevin, V. , Saunders, M. E. , & Rader, R. (2019). Evaluating the taxa that provide shared pollination services across multiple crops and regions. Scientific Reports, 9(1), 1–10. 10.1038/s41598-019-49535-w 31537826 PMC6753147

[ece38653-bib-0106] Williams, P. H. , Ito, M. , Matsumura, T. , & Kudo, I. (2010). The bumblebees of the Nepal Himalaya (Hymenoptera: Apidae). Insecta matsumurana. New Series: Journal of the Faculty of Agriculture Hokkaido University, Series Entomology, 66, 115–151.

[ece38653-bib-0107] Willmer, P. G. (1983). Thermal constraints on activity patterns in nectar‐feeding insects. Ecological Entomology, 8(4), 455–469. 10.1111/j.1365-2311.1983.tb00524.x

[ece38653-bib-0108] Winfree, R. , Griswold, T. , & Kremen, C. (2007). Effect of human disturbance on bee communities in a forested ecosystem. Conservation Biology, 21(1), 213–223. 10.1111/j.1523-1739.2006.00574.x 17298527

[ece38653-bib-0109] Wojcik, V. (2021). Pollinators: Their evolution, ecology, management, and conservation. In Arthropods. IntechOpen. 10.5772/intechopen.97153

