# Supplementary material for: Community structure of pollinating insects and its driving factors in different habitats of Shivapuri‐Nagarjun National Park, Nepal
Source: Ecol Evol. 2022 Mar 1;12(3):e8653. doi: 10.1002/ece3.8653 (PMC8888256; doi:10.1002/ece3.8653)
Supplement: Supplementary file 1 — Supplementary Material 1 [file ECE3-12-e8653-s001.docx]

**Supplementary 1** Family, scientific name and number of each species of insect pollinators collected from Shivapuri-Nagarjun National Park, Nepal

| **Family** | **Genus** | **species** | **Number** | **Author** | **Date** |
| --- | --- | --- | --- | --- | --- |
| **Order Lepidoptera** | | | | | |
| Hesperiidae | *Parnara* | *naso* | 26 | Moore | 1878 |
|  |  | *guttata* | 42 | Bremer & Grey | 1852 |
|  | *Pelopidas* | *agna* | 36 | Moore | 1865 |
|  |  | *mathias* | 40 | Fabricius | 1798 |
| Lycaenidae | *Chrysozephyrus* | *birupa* | 23 | Moore | 1877 |
|  | *Cyrestis* | *thyodamas* | 18 | Boisduval | 1836 |
|  | *Lampides* | *boeticus* | 64 | Linnaeus | 1767 |
|  | *Lestranicus* | *transpectus* | 71 | Moore | 1879 |
|  | *Rapala* | *manea* | 28 | Hewitson | 1863 |
|  | *Udara* | *dilectus* | 45 | Moore | 1879 |
|  | *Zizeeria* | *maha* | 93 | Kollar | 1844 |
| Nymphalidae | *Aglais* | *caschmirensis* | 296 | Kollar | 1844 |
|  | *Appias* | *lyncida* | 11 | Cramer | 1777 |
|  | *Argyreus* | *hyperbius* | 50 | Linnaeus | 1763 |
|  | *Athyma* | *cama* | 14 | Moore | 1858 |
|  |  | *jina* | 27 | Moore | 1858 |
|  |  | *opalina* | 42 | Kollar | 1844 |
|  | *Childrena* | *childreni* | 23 | Gray | 1831 |
|  | *Danaus* | *chrysippus* | 48 | Linnaeus | 1758 |
|  | *Danaus* | *genutia* | 47 | Cramer | 1779 |
|  | *Dichorragia* | *nesimachus* | 3 | Doyère | 1840 |
|  | *Euploea* | *core* | 69 | Cramer | 1780 |
|  |  | *mulciber* | 64 | Cramer | 1777 |
|  | *Fabriciana* | *kamala* | 24 | Moore | 1857 |
|  | *Hypolimnas* | *misippus* | 8 | Linnaeus | 1764 |
|  | *Lethe* | *confusa* | 13 | Aurivillius | 1897 |
|  |  | *insana* | 31 | Kollar | 1844 |
|  |  | *kansa* | 54 | Moore | 1857 |
|  |  | *serbonis* | 23 | Hewitson | 1876 |
|  |  | *verma* | 108 | Kollar | 1844 |
|  |  | *vindhya* | 10 | Felder | 1859 |
|  | *Melanitis* | *phedima* | 32 | Cramer | 1780 |
|  | *Mycalesis* | *francisca* | 18 | Stoll | 1780 |
|  |  | *mineus* | 20 | Linnaeus | 1758 |
|  |  | *perseus* | 32 | Fabricius | 1775 |
|  | *Neptis* | *ananta* | 29 | Moore | 1857 |
|  |  | *hylas* | 58 | Linnaeus | 1758 |
|  |  | *mahendra* | 98 | Moore | 1872 |
|  |  | *manasa* | 13 | Moore | 1857 |
|  | *Orsotriaena* | *medus* | 31 | Fabricius | 1775 |
|  | *Parantica* | *aglea* | 31 | Stoll | 1782 |
|  |  | *pedonga* | 20 | Fujioka | 1970 |
|  | *Phaedyma* | *columella* | 49 | Cramer | 1782 |
|  |  | *almana* | 14 | Linnaeus | 1758 |
|  | *Precis* | *iphita* | 53 | Cramer | 1779 |
|  |  |  |  |  |  |
|  | *Vanessa* | *cardui* | 170 | Linnaeus | 1758 |
|  |  | *indica* | 70 | Herbst | 1794 |
|  | *Ypthima* | *avanta* | 20 | Moore | 1874 |
|  |  | *baldus* | 128 | Fabricius | 1775 |
|  |  | *confusa* | 12 | Shirozu & Shima | 1977 |
|  |  | *hannyngtoni* | 13 | Eliot | 1967 |
|  |  | *indica* | 27 | Hewitson | 1865 |
|  | *Zophoessa* | *goalpara* | 13 | Moore | 1866 |
|  |  | *sidonis* | 22 | Hewitson | 1863 |
| Papilionidae | *Graphium* | *agamemnon* | 22 | Linnaeus | 1758 |
|  |  | *sarpedon* | 43 | Linnaeus | 1758 |
| *Papilio* | *castor* | 71 | Westwood | 1842 |  |
|  | *helenus* | 85 | Linnaeus | 1758 |  |
|  | *krishna* | 3 | Moore | 1857 |  |
|  |  | *memnon* | 66 | Linnaeus | 1758 |
|  |  | *polytes* | 52 | Linnaeus | 1758 |
|  |  | *protenor* | 50 | Cramer | 1775 |
|  |  | *rhetenor* | 47 | Westwood | 1842 |
| Pieridae | *Cepora* | *nadina* | 44 | Lucas | 1852 |
|  | *Colias* | *erate* | 86 | Esper | 1805 |
|  |  | *fieldii* | 87 | Menetries | 1855 |
| *Delias* | *belladonna* | 14 | Fabricius | 1793 |  |
| *Eurema* | *hecabe* | 83 | Linnaeus | 1758 |  |
|  | *Gonepteryx* | *rhamni* | 45 | Linnaeus | 1758 |
|  | *Hebomoia* | *glaucippe* | 7 | Linnaeus | 1758 |
|  | *Metaporia* | *agathon* | 22 | Gray | 1831 |
|  | *Pieris* | *brassicae* | 58 | Linnaeus | 1758 |
|  |  | *canidia* | 255 | Linnaeus | 1768 |
|  | *Catopsilia* | *pomona* | 64 | Fabricius | 1775 |
| Riodinidae | *Abisara* | *fylla* | 72 | Westwood | 1851 |
|  | *Dodona* | *dipoea* | 115 | Hewitson | 1865 |
|  |  | *durga* | 7 | Kollar& Redtenbacher | 1844 |
|  |  | *egeon* | 46 | Westwood | 1851 |
|  | *eugenes* | 169 | Bates | 1868 |  |
| *Zemeros* | *flegyas* | 38 | Cramer | 1780 |  |
| Satyridae | *Dallacha* | *hyagriva* | 21 | Moore | 1858 |
| **Order Diptera** | | | | | |
| Syrphidae | *Eristalinus* | *aeneus* | 21 | Scopoli | 1763 |
|  |  | *arvorum* | 22 | Fabricius | 1787 |
|  |  | *taeniops* | 44 | Wiedemann | 1818 |
|  | *Eristalis* | *cerealis* | 292 | Fabricius | 1805 |
|  |  | *himalayensis* | 29 | Brunetti | 1908 |
|  |  | *tenax* | 260 | Linnaeus | 1758 |
|  | *Graptomyza* | *nigripes* | 21 | Brunetti | 1913 |
|  | *Lycastris* | *albipes* | 19 | Walker | 1857 |
|  |  | *flavohirta* | 16 | Brunetii | 1907 |
|  | *Mesembrius* | *bengalensis* | 28 | Rondani | 1857 |
|  | *Phytomia* | *errans* | 22 | Fabricius | 1787 |
|  |  | *zonata* | 6 | Fabricius | 1787 |
|  | *Rhingia* | *siwalikensis* | 1 | Nayar | 1968 |
|  | *Syritta* | *indica* | 61 | Wiedemann | 1824 |
|  |  | *orientalis* | 61 | Macquart | 1842 |
|  |  | *pipiens* | 65 | Linnaeus | 1758 |
|  | *Volucella* | *trifasciata* | 1 | Wiedemann | 1830 |
|  | *Asarkina* | *porcina* | 22 | Coquillett | 1898 |
|  |  | *incisuralis* | 17 | Macquart | 1855 |
|  | *Baccha* | *maculata* | 5 | Walker | 1852 |
|  | *Betasyrphus* | *serarius* | 11 | Wiedemann | 1830 |
|  | *Dasysyrphus* | *orsua* | 55 | Walker | 1852 |
|  | *Episyrphus* | *balteatus* | 222 | De Geer | 1776 |
|  |  | *viridaureus* | 248 | Wiedemann | 1824 |
|  | *Eupeodes* | *bucculatus* | 84 | Rondani | 1857 |
|  | *Melanostoma* | *orientale* | 44 | Wiedemann | 1824 |
|  |  | *scalare* | 87 | Fabricius | 1794 |
|  |  | *univittatum* | 93 | Wiedemann | 1824 |
|  | *Meliscaeva* | *cinctella* | 50 | Zetterstedt | 1843 |
|  | *Paragus* | *crenulatus* | 37 | Thomson | 1869 |
|  | *Parasyrphus* | sp.1 | 58 |  |  |
|  | *Scaeva* | *pyrastri* | 14 | Linnaeus | 1758 |
|  | *Sphaerophoria* | *bengalensis* | 59 | Macquart | 1842 |
|  |  | *indiana* | 71 | Bigot | 1884 |
|  |  | *scripta* | 35 | Linnaeus | 1758 |
|  | *Syrphus* | *torvus* | 47 | Osten Sacken | 1875 |
| **Order Hymenoptera** | | | | | |
| Andrenidae | *Andrena* | *flavipes* | 77 | Panzer | 1799 |
|  |  | *gorkhana* | 58 | Tadauchi & Matsumura | 2007 |
|  |  | *kathmanduensis* | 34 | Tadauchi & Matsumura | 2007 |
| Apidae | *Ceratina* | *bryanti* | 63 | Cockerell | 1919 |
|  |  | *dentipes* | 36 | Friese | 1914 |
|  |  | *perforatrix* | 58 | Smith | 1879 |
|  |  |  |  |  |  |
|  | *Nomada* | *mutabilis* | 4 | Morawitz | 1871 |
|  |  | sp.1 | 3 |  |  |
|  | *Thyreus* | *decorus* | 16 | Smith | 1852 |
|  |  | *himalayensis* | 14 | Radoszkowski | 1893 |
|  |  | *histrio* | 19 | Fabricius | 1775 |
|  | *Xylocopa* | *acutipennis* | 25 | Smith | 1854 |
|  |  | *aestuans* | 51 | Linnaeus | 1758 |
|  |  | *collaris* | 39 | Lepeletier | 1841 |
|  |  | *fenestrata* | 47 | Fabricius | 1798 |
|  |  | *tenuiscapa* | 48 | Westwood | 1840 |
|  |  | *violacea* | 11 | Linnaeus | 1758 |
|  | *Amegilla* | *candida* | 26 | Smith | 1879 |
|  |  | *cingulifera* | 42 | Cockerell | 1910 |
|  |  | *confusa* | 20 | Smith | 1854 |
|  |  | *fallax* | 32 | Smith | 1879 |
|  |  | *insularis* | 33 | Smith | 1857 |
|  |  | *quadrifasciata* | 37 | de Villers | 1789 |
|  |  | *zonata* | 37 | Linnaeus | 1758 |
|  | *Elaphropoda* | *impatiens* | 35 | Lieftinck | 1944 |
|  | *Apis* | *cerana* | 386 | Fabricius | 1793 |
|  |  | *mellifera* | 146 | Linnaeus | 1761 |
|  | *Bombus* | *breviceps* | 5 | Smith | 1852 |
|  |  | *eximius* | 95 | Smith | 1852 |
|  |  | *flavescens* | 102 | Smith | 1852 |
|  |  | *haemorrhoidalis* | 128 | Smith | 1852 |
| Colletidae | *Colletes* | *similis* | 55 | Schenck | 1853 |
|  | sp.1 | 29 |  |  |  |
|  | sp.2 | 22 |  |  |  |
| Halictidae | *Halictus* | *intricatus* | 27 | Vachal | 1894 |
|  |  | *propinquus* | 19 | Smith | 1853 |
|  |  | *senescens* | 54 | Smith | 1879 |
|  |  | *subopacus* | 40 | Smith | 1853 |
|  |  | *vicinus* | 34 | Vachal | 1895 |
|  | *Lasioglossum* | *albescens* | 51 | Smith | 1853 |
|  |  | *albipes* | 50 | Fabricius | 1781 |
|  |  | sp*.*1 | 49 |  |  |
|  |  | *marginatum* | 54 | Brullé | 1853 |
|  |  | *villosulum* | 34 | Kirby | 1802 |
|  | *Nomia* | *formosa* | 33 | Smith | 1858 |
|  |  | *incerta* | 39 | Gribodo | 1894 |
|  |  | *iridescens* | 30 | Smith | 1858 |
| Megachilidae | *Megachile* | *conjuncta* | 17 | Smith | 1853 |
|  |  | *femorata* | 13 | Smith | 1853 |
|  |  | *umbripennis* | 19 | Smith | 1853 |
