# Supplementary material for: Community structure of pollinating insects and its driving factors in different habitats of Shivapuri‐Nagarjun National Park, Nepal
Source: Ecol Evol. 2022 Mar 1;12(3):e8653. doi: 10.1002/ece3.8653 (PMC8888256; doi:10.1002/ece3.8653)
Supplement: Supplementary file 2 — Supplementary Material 2 [file ECE3-12-e8653-s003.docx]

**Supplementary 2** Herbs and shrubs present in the sampling transects of Shivapuri-Nagarjun National Park, Nepal

| **Flower** |
| --- |
| *Ageratina adenophora* |
| *Ageratum conyzoides* |
| *Ageratum houstonianum* |
| *Astragalus khasianus* |
| *Bidens pilosa* |
| *Campanula argyrotricha* |
| *Porana racemosa* |
| *Crepis* sp. |
| *Drymaria cordata* |
| *Eclipta prostrata* |
| *Galinsoga ciliate* |
| *Galinsoga parviflora* |
| *Gaultheria fragrantissima* |
| *Geranium nepalense* |
| *Gnaphalium hypoleucum* |
| *Hypericum japonicum* |
| *Indigofera* sp*.* |
| *Lantana camara* |
| *Leucas cephalotes* |
| *Myrsine capitellata* |
| *Osbeckia stellate* |
| *Oxalis corniculata* |
| *Oxyspora paniculata* |
| *Parthenium hysterophorus* |
| *Persicaria capitata* |
| *Persicaria* sp*.* |
| *Ranunculus* sp. |
| *Rubus ellipticus* |
| *Senna alexandrina* |
| *Sida rhombifolia* |
| *Taraxacum officinale* |
| *Trifolium repens* |
| *Viburnum cylindricum* |
| *Viola serpens* |
| *Achyranthes aspera* |
| *Aconogonum molle* |
| *Alternanthera sessilis* |
| *Anaphalis busua* |
| *Artemisia dubia* |
| *Tridax procumbens* |
| *Bupleurum hamiltonii* |
| *Carex felicina* |
| *Cerastium holosteoides* |
| *Coriaria nepalensis* |
| *Daphne bholua* |
| *Elsholtzia strobilifera* |
| *Fagopyrum esculentum* |
| *Fagopyrum tataricum* |
| *Guizotia abyssinica* |
| *Hedychium spicatum* |
| *Justicia simplex* |
| *Luculia gratissima* |
| *Plectranthus barbatus* |
| *Polygonum hydropiper* |
| *Reinwardtia indica* |
| *Sarcococca wallichii* |
| *Scutellaria* sp. |
| *Strobilanthes atropurpureus* |
| *Swertia angustifolia* |
| *Urena lobate* |
