# Supplementary material for: Community structure of pollinating insects and its driving factors in different habitats of Shivapuri‐Nagarjun National Park, Nepal
Source: Ecol Evol. 2022 Mar 1;12(3):e8653. doi: 10.1002/ece3.8653 (PMC8888256; doi:10.1002/ece3.8653)
Supplement: Supplementary file 3 — Supplementary Material 3 [file ECE3-12-e8653-s002.docx]

**Supplementary 3** Range of flower resource abundance in different habitats in two seasons along the elevation gradient of Shivapuri-Nagarjun National Park, Nepal

| Habitats | Elevation (meter) | Flower resource abundance range | |
| --- | --- | --- | --- |
|  |  | Autumn | Spring |
| Forest trail | 1500 – 1700 | 26–33 | 27–38 |
|  | 1800 – 1900 | 20–32 | 25–29 |
|  | 2000 – 2700 | 8–24 | 14–28 |
| Grass land | 1500 – 1700 | 17–21 | 13–16 |
|  | 1800 – 1900 | 16–21 | 12–16 |
|  | 2000 – 2700 | 18–20 | 14–16 |
| Open trail | 1500 – 1700 | 72–88 | 84–87 |
|  | 1800 – 1900 | 71–88 | 84–88 |
|  | 2000 – 2700 | 26–88 | 29–88 |
| Managed habitat | 1500 – 1700 | 72–84 | 71–74 |
|  | 1800 – 1900 | 75–81 | 72–73 |
|  | 2000 – 2700 | 69–83 | 64–74 |
